# 

**Published:** 1893-01

**Authors:** 


### CONTENTS.

COMMUNICATIONS.

Address of President of the Ohio State Dental Society .......... 1
The Breath. .......... 5
Mechanical Abrasion of the Teeth .......... 12
Obtunding Sensitive Dentine .......... 16
Present and Future of Prosthetic Dentistry .......... 18
Sterilization of the Hands and Instruments of Dentists .......... 22
How to Practice Dentistry Successfully .......... 26
An Unclassified Disease .......... 30
Sarcasm .......... 30

SELECTIONS.

Waste Products made Useful .......... 31
Safe and Perilous Occupations .......... 41
Culture and Professional Success .......... 44
Light as a Caustic .......... 47

EDITORIAL.

Ohio State Dental Examining Board .......... 48
Meeting of the National Association of Dental Faculties for 1893 .......... 49
Alumni Meetings .......... 50
Ohio State Dental Society .......... 50
Bibliographical .......... 51

